# Native Top-Down Mass Spectrometry Uncovers Two Distinct Binding Motifs of a Functional Neomycin-Sensing Riboswitch Aptamer

**DOI:** 10.1021/jacs.3c02774

**Published:** 2023-07-07

**Authors:** Sarah Viola Heel, Karolina Bartosik, Fabian Juen, Christoph Kreutz, Ronald Micura, Kathrin Breuker

**Affiliations:** Institute of Organic Chemistry and Center for Molecular Biosciences Innsbruck (CMBI), University of Innsbruck, Innrain 80/82, 6020 Innsbruck, Austria

## Abstract

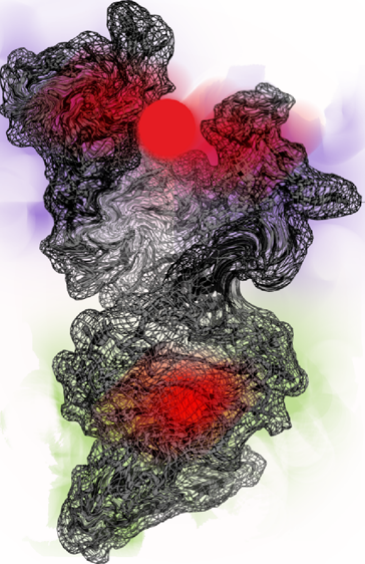

Understanding how ligands bind to ribonucleic acids (RNA) is important for understanding RNA recognition in biological processes and drug development. Here, we have studied neomycin B binding to neomycin-sensing riboswitch aptamer constructs by native top-down mass spectrometry (MS) using electrospray ionization (ESI) and collisionally activated dissociation (CAD). Our MS data for a 27 nt aptamer construct reveal the binding site and ligand interactions, in excellent agreement with the structure derived from nuclear magnetic resonance (NMR) studies. Strikingly, for an extended 40 nt aptamer construct, which represents the sequence with the highest regulatory factor for riboswitch function, we identified two binding motifs for neomycin B binding, one corresponding to the bulge-loop motif of the 27 nt construct and the other one in the minor groove of the lower stem, which according to the MS data are equally populated. By replacing a noncanonical with a canonical base pair in the lower stem of the 40 nt aptamer, we can reduce binding to the minor groove motif from ∼50 to ∼30%. Conversely, the introduction of a CUG/CUG motif in the lower stem shifts the binding equilibrium in favor of minor groove binding. The MS data reveal site-specific and stoichiometry-resolved information on aminoglycoside binding to RNA that is not directly accessible by other methods and underscore the role of noncanonical base pairs in RNA recognition by aminoglycosides.

## Introduction

To better understand biological processes that involve ribonucleic acids (RNA) and to advance the development of potential therapeutics that target RNA,^1−5^ it is essential to understand how ligands bind to RNA in terms of affinity and specificity. Previous studies found that high-affinity binding is not necessarily highly specific, and low-affinity binding is not necessarily unspecific. For example, RNA aptamers selected for higher-affinity binding did not show higher specificity for the guanosine triphosphate ligand.^6^ Likewise, neomycin B binds to the HIV-1 rev response element (RRE) stem II RNA with ∼100-fold higher affinity than kanamycin A, but the specificity of binding to RRE stem II RNA is higher for kanamycin A than for neomycin B.^7,8^ Tor and coworkers have even suggested that the inverse correlation between binding affinity and specificity observed in aminoglycoside and aminoglycoside derivative binding to RRE stem II RNA may represent a general principle of small molecule-RNA binding.^7^ Apparently, ligand binding to RNA is far more intricate than what a simple model would predict and could involve more than one binding motif.^9−13^

Aminoglycosides can bind to many different types of RNA, including ribosomal RNA, viral RNA, transfer RNA, and riboswitches, which is why they have been described as excellent model ligands for the study of RNA recognition.^11,13−17^ The interactions of aminoglycosides with RNA are largely electrostatic in nature due to the positive charge of the aminoglycosides and the negative charge of the RNA at neutral and near-neutral pH but can be highly specific when the binding interface includes networks of directional intermolecular interactions such as hydrogen bonds.^15,16,18−20^ Numerous studies have investigated RNA-aminoglycoside interactions using a variety of methods, including nuclear magnetic resonance (NMR) spectroscopy,^9,18,21−28^ crystallography,^12,14,29−31^ and enzymatic or chemical probing approaches.^15,32−34^ The majority of NMR and crystal structures of RNA-aminoglycoside complexes show aminoglycoside binding to various motifs formed by nucleotides in and next to internal loops or bulged regions,^15^ and noncanonical base pairs were suggested to be important for recognition by the aminoglycoside ligand.^35^ However, since aminoglycosides are conformationally flexible and can adopt to RNA structures, which themselves can be highly dynamic,^36^ it is challenging to predict binding affinity and specificity.^37^ To learn more about RNA-aminoglycoside interactions, Disney and coworkers developed a two-dimensional combinatorial screening method for the identification of high-affinity, specific interactions between aminoglycosides and model RNAs with various hairpin and internal loop motifs.^38−42^ More recently, Hargrove and coworkers used data from aminoglycoside binding to model RNAs with different secondary structure motifs (hairpin loops, symmetric and asymmetric internal loops, bulges, and stems) and principal component analysis to develop an unbiased approach for the classification of RNA structures.^16,43,44^

A method that can complement the above techniques and does not require isotope labeling, crystallization, enzymes, or chemical reagents is native mass spectrometry (MS). Since the late 1990s, RNA-protein and RNA-drug complexes have been studied by native electrospray ionization (ESI) MS, which can directly reveal the identity of RNA binding partners and the stoichiometry of the complexes present in solution.^45−61^ For example, in separate native ESI MS studies, Loo et al., Hofstadler et al., and Fabris and Hagan observed RNA-aminoglycoside complexes with higher stoichiometry, which they interpreted as either unspecific binding or low-affinity binding to sites other than those of the 1:1 complexes.^49,52,62^ Inspired by our recent studies of RNA-peptide complexes,^63,64^ we wondered if we could use native ESI MS in combination with low-energy collisionally activated dissociation (CAD)^65,66^ to contribute to a better understanding of how small molecules interact with RNA, especially with respect to specific versus unspecific binding and multiple binding motifs. A major advantage of native ESI combined with CAD is that this approach can be used to characterize wild-type RNA-ligand complexes even when their conformational dynamics complicate the interpretation of NMR data^21,67^ or interfere with RNA crystallization.^68−70^ As a model system for our studies, we chose 40 nt aptamer constructs based on the sequence of a synthetic neomycin-sensing riboswitch, which showed high ligand specificity for neomycin B and dose-dependent regulation of gene expression.^71,72^ Moreover, we have studied a 27 nt aptamer construct of the neomycin-sensing riboswitch for whose complex with paromomycin, an NMR structure, is available.^73^

## Results and Discussion

**Figure 1:**
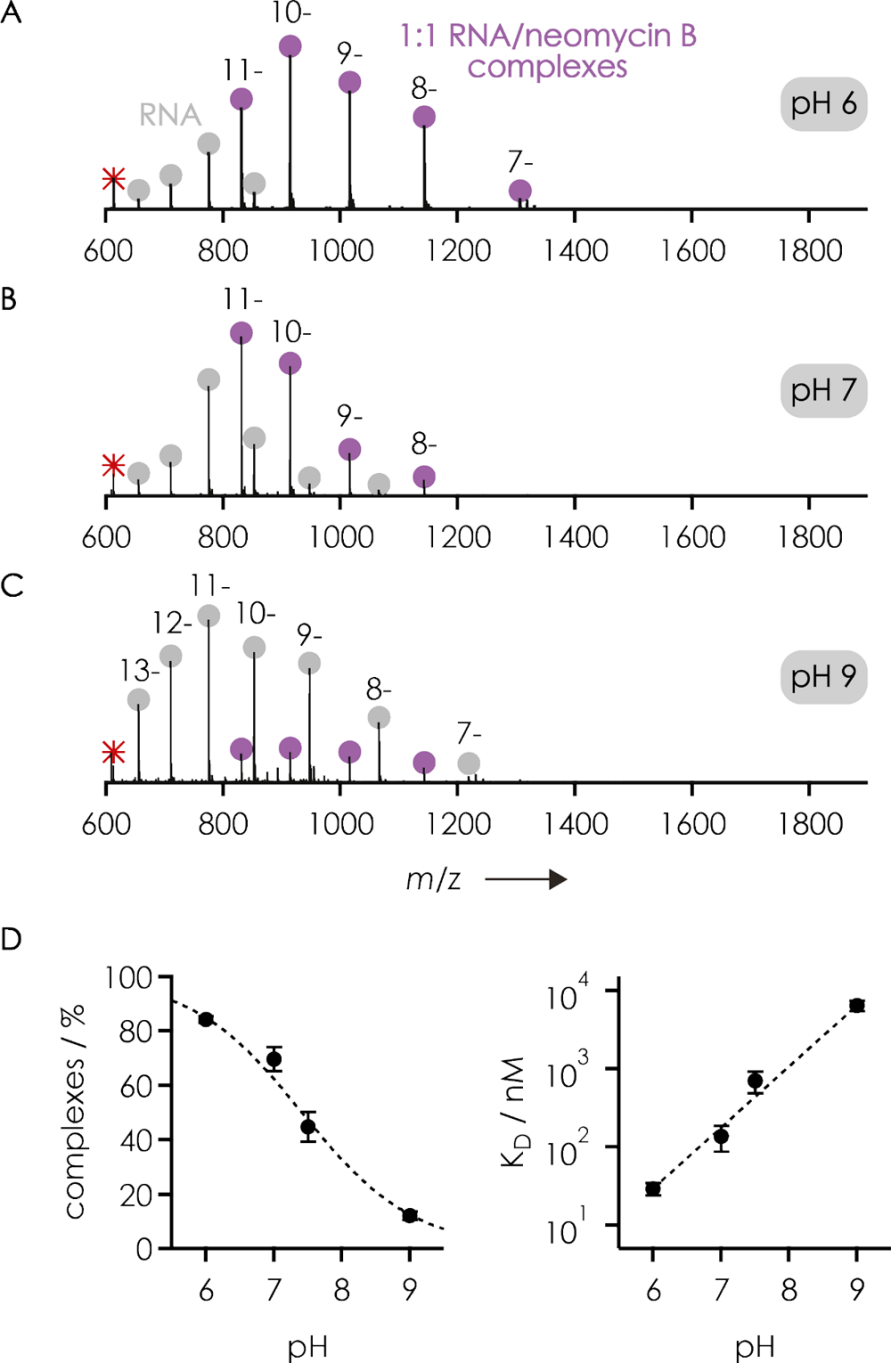
Native ESI MS spectra of equimolar solutions of the 27 nt neomycin-sensing riboswitch aptamer RNA **1** and neomycin B (1 μM each) in 9:1 H_2_O/CH_3_OH at (A) pH ∼6 (with 0.25 mM piperazine as an ESI additive), (B) pH ∼7 (2 mM piperazine), and (C) pH ∼9 (2 mM piperidine) incubated for 3 h at room temperature (21 °C) showing signals of 1:1 complexes (violet), free RNA (gray), and neomycin B (red). (D) Effect of solution pH on the fraction of complexes (left) and the corresponding *K*_D_ values (right) calculated according to method 1, which assumes a single binding site as described in the Supporting Information; shown are average values from triplicate experiments with error bars representing the standard deviation. Data at pH 7.5 are from ESI of solutions with 50 mM ammonium bicarbonate and 0.25 mM piperazine, and dashed lines are meant to guide the eyes.

Figure 1A–C shows spectra from native ESI of equimolar solutions of a 27 nt aptamer construct of the neomycin-sensing riboswitch used in NMR studies (RNA **1**, Table 1) and neomycin B, with signals for 1:1 complexes, free RNA, and the free ligand. The fraction of RNA complexes was 84 ± 1% at pH ∼6, 70 ± 4% at pH ∼7, 45 ± 6% at pH 7.5, and 12 ± 2% at pH ∼9 (Figure 1D), consistent with a decrease in neomycin B protonation^74−77^ with increasing pH, which in turn decreases the number of electrostatic interactions essential for aminoglycoside binding to the RNA.^12,17,78,79^ The *K*_D_ values calculated from the fractions of RNA complexes in the spectra from ESI at room temperature (21 °C) and assuming a single binding site^62^ (model 1 in the Supporting Information) were 29 ± 5 nM at pH ∼6, 0.14 ± 0.05 μM at pH ∼7, 0.7 ± 0.2 μM at pH 7.5, and 6 ± 1 μM at pH ∼9 (Figure 1D), all of which are lower than the *K*_D_ of 10 ± 2 nM at pH 6.8 and 37 °C determined by isothermal titration calorimetry (ITC) by Wöhnert and coworkers.^73^ Data from ITC experiments at 21 °C with solutions closely resembling those for ESI (with the RNA concentration increased to 8–10 μM for reasons of sensitivity) were not suitable for the determination of *K*_D_ values even though imino proton spectra from nuclear magnetic resonance (NMR) clearly showed that the RNA was folded (Figure S1). We then conducted ITC experiments at pH 7.5 and 21 °C using a buffer typical for ITC of RNA.^80^ The ITC data indicate two binding sites for RNA **1** (Figure S2) with *K*_D_ values (2 ± 2 nM and 0.6 ± 0.2 μM) that differ by ∼2.4 orders of magnitude, meaning that in 1:1 mixtures of RNA **1** with neomycin B, the latter is almost exclusively bound to the high-affinity site. Fitting the ITC data with a single-site model instead results in a *K*_D_ value (0.7 ± 0.2 μM) that matches the *K*_D_ value from ESI at the same pH (0.7 ± 0.2 μM, also assuming a single binding site). Higher concentrations of the aminoglycoside ligand were required to observe similar fractions of 1:1 complexes of RNA **1** and paromomycin by native ESI MS. For a solution with RNA **1** (1 μM) and paromomycin (10 μM) at pH ∼6 and room temperature (Figure S3), the ESI data showed 67 ± 6% RNA complexes, which indicates a *K*_D_ of 4.6 ± 1.4 μM, in good agreement with the *K*_D_ of 5.13 ± 0.26 μM from ITC measurements (at pH 6.8 and 37 °C).^73^ We conclude that although native ESI MS^52,54−56,58,62^ and ITC^78,81−83^ are conceptually different, ESI MS spectra can be used for the calculation of *K*_D_ values that agree well with those from ITC, provided that a single-site model adequately describes the binding equilibrium. For systems with more than one binding site, the ESI MS data can at least provide an estimate of the overall binding affinity. However, without site-specific binding information, it is generally difficult to distinguish between single and multiple binding sites.^9^ To further investigate the neomycin-sensing riboswitch aptamer and to address this issue by determining the sites of neomycin B and paromomycin binding, we used our recently developed MS approach with ion isolation in the quadrupole followed by collisionally activated dissociation (CAD) in the collision cell of a Fourier transform ion cyclotron resonance (FT-ICR) instrument.^64,65^

**Table 1:** RNA Studies (All OH-Terminated)^a^

RNA	sequence	nt
**1**	5′- GGCU**GCUUGUCCUUUAAUGGUCC**AGUC-3′	27
**2**	5′-CCGGCAUA**GCUUGUCCUUUAAUGGUCC**UAUGUCGAAAAUG-3′	40
**3**	5′-CCGUCAUA**GCUUGUCCUUUAAUGGUCC**UAUGACGAAAAUG-3′	40
**4**	5′-CCCUGAUA**GCUUGUCCUUUAAUGGUCC**UAUCUGGAAAAUG-3′	40

a Conserved sequence^71^ in bold.

**Figure 2:**
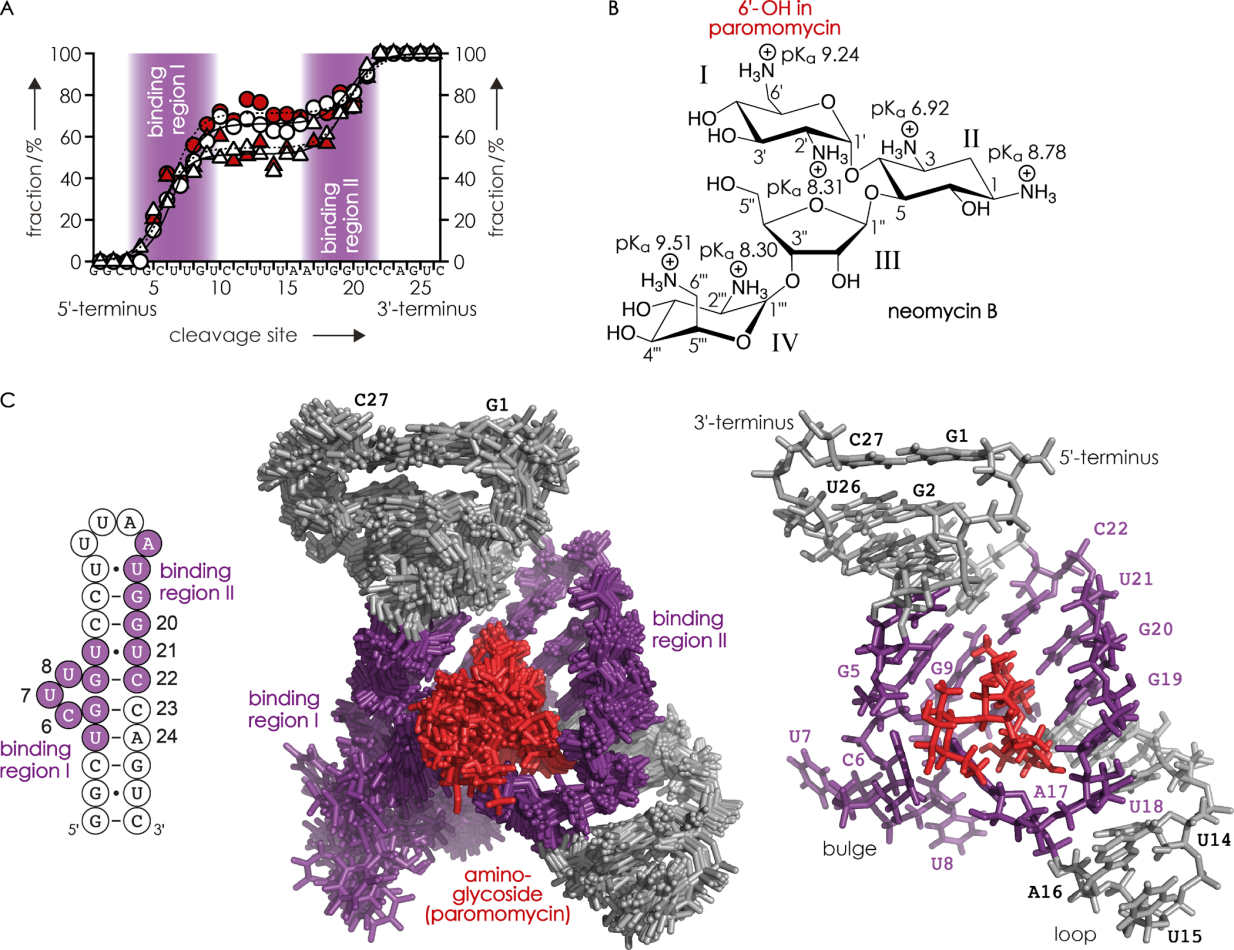
(A) Fraction of ***c*** fragments (circles) with neomycin B (white) or paromomycin (red) and complementary ***y*** fragments (triangles) without neomycin B (white) or paromomycin (red) attached from CAD (103.5 eV laboratory frame energy) of 1:1 complexes of the neomycin-sensing riboswitch with neomycin B or paromomycin, (RNA **1** + neomycin B - 9H)^9–^ or (RNA **1** + paromomycin - 9H)^9–^, from ESI of solutions at pH ∼6, versus the RNA cleavage site; lines are double sigmoidal fits meant to guide the eyes (solid for neomycin B and dashed for paromomycin); (B) chemical structure of neomycin B with p*K*_a_ values of amino groups from ref (74); (C) neomycin B and paromomycin binding regions I and II derived from CAD data (violet) mapped onto the NMR structure 2MXS of the neomycin-sensing riboswitch in complex with paromomycin (left: secondary structure, center: all 20 structure models, and right: model 1); the aminoglycoside is highlighted in red.

Isolation and CAD of the 1:1 complexes of the 27 nt aptamer construct of the neomycin-sensing riboswitch with neomycin B and a net charge of 9–, (RNA **1** + neomycin B - 9H)^9–^, at *m*/*z* ∼1016 produced ***c*** and ***y*** fragments from phosphodiester backbone bond cleavage^84,85^ with and without neomycin B attached and only ∼1% free RNA from neomycin B dissociation (relative to undissociated complexes), which shows that the electrostatic interactions between neomycin B and RNA **1** are sufficiently strong for binding site mapping by CAD.^63−65^Figure 2A illustrates the fraction of ***c*** fragments with neomycin B attached (white circles) and the fraction of complementary ***y*** fragments without neomycin B attached (white triangles) plotted against the RNA cleavage site. The below data interpretation for the identification of ligand binding regions of RNA^63,64^ is conceptually the same as for nucleobase modifications^34,86^ and considers site-specific fractions of ***c*** and ***y*** fragments with and without neomycin B attached. No neomycin B was found on fragments ***c***_1_–***c***_3_, and no complementary fragments ***y***_26_–***y***_24_ without neomycin B were observed, indicating that neomycin B does not bind to G1-C3. The fraction of ***c*** fragments with neomycin B attached sigmoidally increased from site 3 to site 10, along with a corresponding increase in the fraction of complementary ***y*** fragments without neomycin B, both of which indicate binding of neomycin B to U4–U10 (binding region I). After a plateau at sites 11–16, which indicates no binding of neomycin B to C11-A16, the fraction of ***c*** fragments with neomycin B and ***y*** fragments without neomycin B further increased to 100% at site 22, which indicates binding of neomycin B to A17-C22 (binding region II). Finally, the 100% values for ***c*** fragments with neomycin B and ***y*** fragments without neomycin B at sites 23–26 are consistent with no binding of neomycin B to C23–C27. The fraction of ***c*** fragments with neomycin B and the complementary ***y*** fragments without neomycin B differ by up to ∼15% in the plateau region, which can be attributed to a higher stability of ***c*** and ***y*** fragments with neomycin B attached against secondary backbone cleavage compared to fragments without a ligand.^63,64^ CAD of the 1:1 complexes of the neomycin-sensing riboswitch with paromomycin, (RNA **1** + paromomycin - 9H)^9–^, also at *m*/*z* ∼1016 and using the same energy, produced very similar data (Figure 2A).

A structure of the neomycin-sensing riboswitch RNA in complex with neomycin B is not yet available, but a solution NMR structure with the very similar paromomycin (PDB entry 2MXS) has been published.^73^ Neomycin B differs from paromomycin only by the presence of an aminium group instead of a hydroxyl group at carbon 6′ of ring I (Figure 2B), and NMR data indicate that the position and orientation of both aminoglycosides in the riboswitch binding pocket as well as the average RNA structure of the complexes are virtually identical.^73^ Our CAD MS data for neomycin B and paromomycin binding to the riboswitch show good agreement with the NMR structure 2MXS (Figure 2C) and strongly support the hypothesis that neomycin B and paromomycin bind to the neomycin-sensing riboswitch in a very similar manner as suggested by NMR^73,87^ and chemical probing MS^34^ data.

CAD of the 1:1 complexes of the 27 nt RNA **1** with neomycin B and a net charge of 9–, (RNA **1** + neomycin B - 9H)^9–^, from ESI of solutions at pH ∼6, ∼7, and ∼9 produced very similar data (Figure S4), which suggests that the pH of the solution only affects the fraction of RNA complexes (Figure 1) but not their structure or the aminoglycoside protonation pattern in the binding interface. The p*K*_a_ values^74^ in Figure 2B indicate that only a marginal fraction of neomycin B is fully protonated at pH 9, but the p*K*_a_ values in the 1:1 complexes are likely substantially higher.^88−90^ In further support of this hypothesis, the average charge of 1:1 complexes electrosprayed from solutions at pH ∼6 and ∼9 was highly similar (9.42 and 9.37, respectively), even though their fraction decreased from 84% at pH ∼6 to 12% at pH ∼9 (Figure 1). Since the NMR data for the structure 2MXS (Figure 2C) were recorded from solutions at higher ionic strength (25 mM potassium phosphate and 50 mM potassium chloride)^73^ than those for ESI, we also investigated the effect of adding 50 mM ammonium bicarbonate to the solution used for ESI. Ion yields were generally lower (by a factor of ∼2) compared to those from solutions without ammonium bicarbonate, but the data from CAD of the 1:1 complexes showed no significant differences (Figure S4B). Further, varying the energy used for CAD of the (RNA **1** + neomycin B - 9H)^9–^ ions from ESI of solutions at pH ∼7 between 99 and 108 eV had no significant effect on the percentage of ***c*** and ***y*** fragments with and without neomycin B, respectively (Figure S5), but CAD at lower energies resulted in signal-to-noise ratios for the less abundant fragments that were too small for reliable relative quantification of fragments with and without neomycin B attached.

Because the aminoglycoside ligand interacts with both binding regions I and II of the riboswitch (Figure 2C), vibrational activation by CAD must not only have cleaved phosphodiester backbone bonds to produce ***c*** and ***y*** fragments^85^ but must also have dissociated some of the noncovalent bonds between the aminoglycoside ligand and the RNA, leaving it bound to either the ***c*** or the complementary ***y*** fragment. The plateaus for ***c*** fragments with and ***y*** fragments without an aminoglycoside ligand in Figure 2A were >50%, which suggests that for the gaseous (RNA **1** + neomycin B - 9H)^9–^ and (RNA **1** + paromomycin - 9H)^9–^ ions, the interactions of paromomycin and neomycin B with binding region I are somewhat more stable than those with binding region II. Data from CAD of (RNA **1** + neomycin B - 10H)^10–^ ions were similar to those of the (RNA **1** + neomycin B - 9H)^9–^ ions (Figure S6) but more scattered because competitive dissociation into ***a*** and ***w*** fragments, which is common in CAD of RNA ions with a higher net negative charge,^91,92^ resulted in lower signal-to-noise ratios for ***c*** and ***y*** fragments.

**Figure 3:**
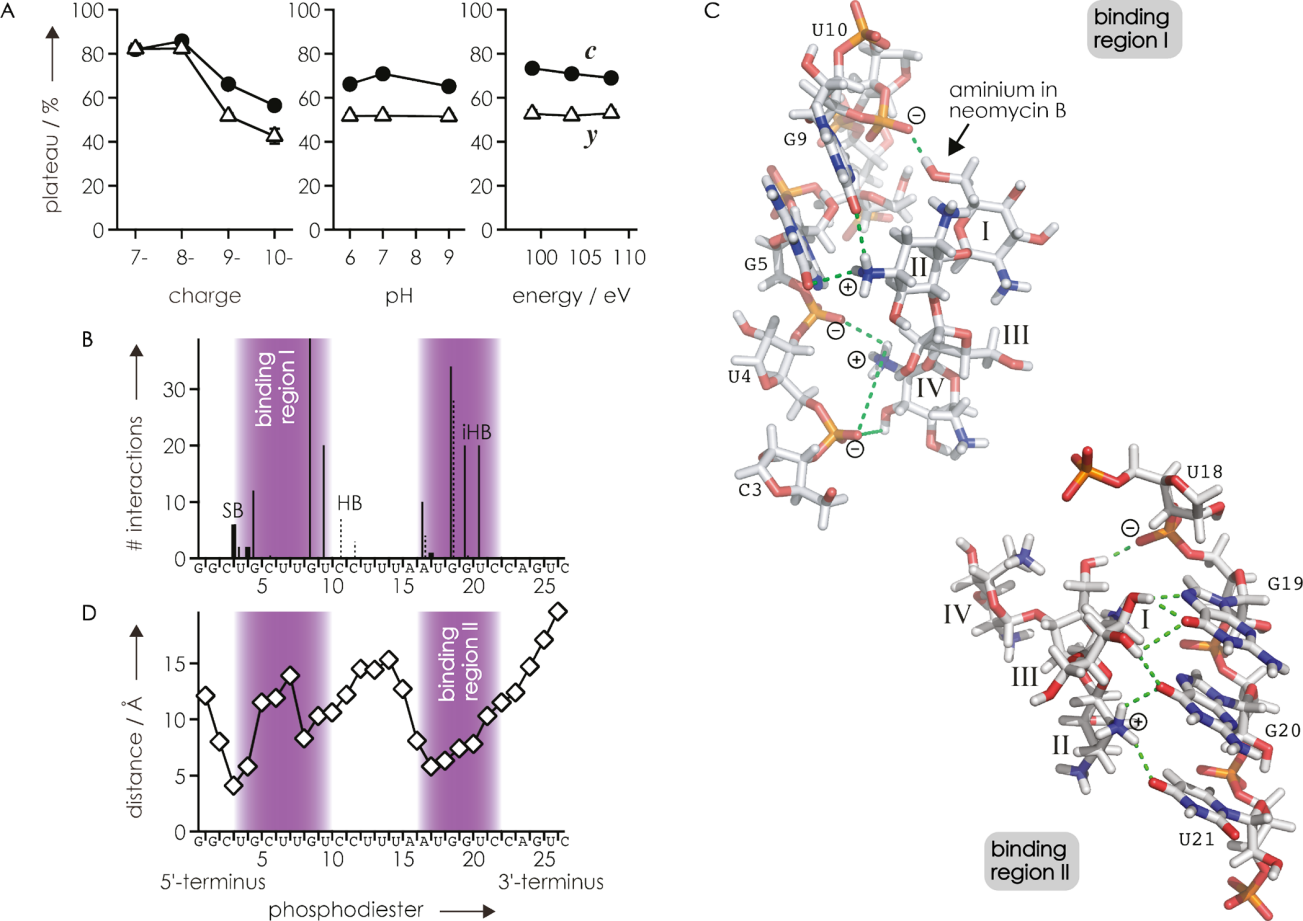
(A) Plateau values for ***c*** (circles) and ***y*** (triangles) fragments versus (RNA **1** + neomycin B - *n*H)^*n*−^ ion net charge, pH (for *n* = 9, 103.5 eV), and energy used for CAD (for *n* = 9, pH ∼7); (B) salt bridge (SB, thick lines), ionic hydrogen bond (iHB, thin lines), and hydrogen bond (HB, dashed lines) interactions between RNA **1** and paromomycin found in the 20 models of the NMR structure 2MXS are clustered in binding regions I and II; (C) selected SBs, iHBs, and HBs (green) in binding regions I and II of the structure 2MXS illustrate networks of electrostatic interactions; (D) distances between phosphorus atoms of the RNA and the nearest nitrogen atoms of paromomycin in the structure 2MXS (model 1).

Figure 3A summarizes that for the (RNA **1** + neomycin B - 9H)^9–^ ions, the plateau values were not affected by solution pH (Figure S4) or the energy used for CAD (Figure S5), but the plateau values increased to ∼80% for the complexes with a net charge of 8– and 7– (Figure S6). This observation is consistent with an increased number of protons that can be mobilized during CAD^85^ in the complexes with a net charge of 8– and 7– and preferential proton transfer to negatively charged sites^64−66,93,94^ in binding region II, which substantially weakens the interactions between binding region II and the aminoglycoside ligand (Figure 3B). Moreover, for the complexes with a net charge of 7–, the CAD data in Figure S6 indicate proton transfer to the phosphates of U4 or G5, thereby disrupting the corresponding salt bridge interactions (Figure 3B) and shifting the first sigmoidal transition of binding region I toward the apparently less susceptible interactions of G9 (Figure 3C). By studying complex ions with a different net charge, we can thus follow the sequential disruption of key electrostatic interactions. Consistent with previous studies,^93−95^ the CAD data in Figure 2A and Figure S6 indicate that the most stable interactions in the gaseous complexes of the neomycin-sensing riboswitch RNA with neomycin B or paromomycin are salt bridges and ionic hydrogen bonds (Figure 3B), which according to the NMR structure 2MXS are part of hydrogen bond networks (Figure 3C) that provide binding specificity. Further, the higher stability of interactions in binding region I compared with binding region II in the gaseous 1:1 complexes (Figure 2A and Figure S6) shows that dissociation by CAD is not governed by simple Coulombic interactions as the distances between the phosphorus atoms of the RNA phosphodiester moieties and the nearest nitrogen atom of paromomycin in the NMR structure would predict an overall higher stability of aminoglycoside interactions with region II compared with region I (Figure 3D). From the above discussion, we conclude that the data from CAD of the 1:1 complexes of the neomycin-sensing riboswitch aptamer with neomycin B (Figure 2A and Figures S4–S6) agree well with the NMR data (Figures 2C and 3B,C) and thus reflect the specific binding of this aminoglycoside to the riboswitch aptamer construct RNA **1**.

**Figure 4:**
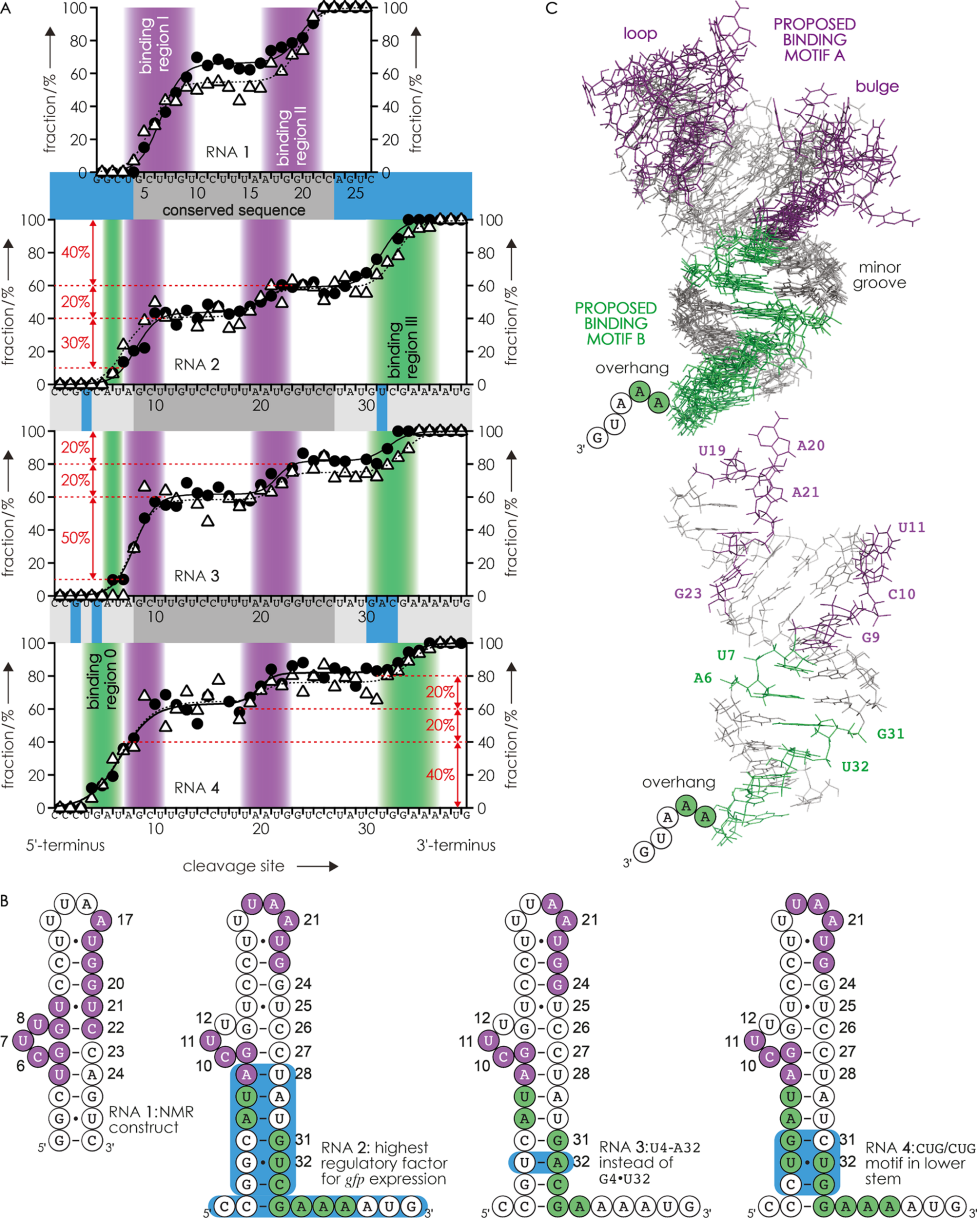
(A) Fraction of ***c*** fragments with neomycin B (circles) and complementary ***y*** fragments without neomycin B (triangles) attached from CAD (137–150 eV laboratory frame energy) of 1:1 complexes of RNA **2**, **3**, and **4** with neomycin B, (RNA + neomycin B - 13H)^13–^, electrosprayed from equimolar solutions in 9:1 H_2_O/CH_3_OH at pH ∼7.5 (1 μM RNA, 1 μM neomycin B, 0.25 mM piperazine, and 50 mM ammonium bicarbonate) versus RNA cleavage site; lines are triple sigmoidal fits and data for RNA **1** (from Figure 2A) are shown for comparison; (B) secondary structure of RNA **1** (from NMR data) and those proposed for RNAs **2**–**4** with CAD-derived neomycin B binding regions I and II in violet and binding regions 0 and III in green; highlighted in blue are residues that differ between the RNA constructs **1**–**4**; (C) structures of RNA **2** (excluding A36-G40) predicted by the MC-fold|MC-Sym pipeline^96^ with binding regions I and II in violet and 0 and III in green (top: 7 representative structure models, bottom: single structure model).

However, the sequence of the 27 nt aptamer construct RNA **1** designed and optimized for the NMR studies^73^ only partially matches the sequence of the 40 nt aptamer construct RNA **2** (Table 1) that we have studied and that is identical to the sequence with the highest regulatory factor for riboswitch function.^71^ CAD of (RNA **2** + neomycin B - 13H)^13–^ ions, with a similar number of net charges per nucleotide (0.325 charges/nt) to the (RNA **1** + neomycin B - 9H)^9–^ ions (0.333 charges/nt) (Figure 2A), indicates small shifts of binding regions I and II in RNA **2** when compared with RNA **1** and two new binding regions 0 and III (Figure 4A). The latter binding regions are located in the extended lower stem and overhang of RNA **2** (Figure 4B), which together with the structure of free RNA **2** predicted by the MC-fold|MC-Sym pipeline^96^ can be interpreted as neomycin B binding to the minor groove (Figure 4C) and stabilization of this binding motif by interactions with the first nucleotides (A35–A37) of the overhang.

Based on the heights of the transitions for RNA **2** of ∼10, ∼30, ∼20, and ∼40% for binding regions 0, I, II, and III, respectively (Figure 4A), it can be estimated that neomycin B binds to the minor groove and the first dangling nucleotides of the overhang of RNA **2** (regions 0 and III, proposed binding motif B) in ∼50% of the complexes and to the binding pocket formed by the loop and the bulge of RNA **2** (regions I and II, proposed binding motif A) in the other ∼50% of the complexes. This result is in line with ITC data (Figure S2) that indicate two binding sites for RNA **2** with *K*_D_ values of comparable magnitude (0.4 ± 0.6 and 3.1 ± 1.5 μM). Replacing the noncanonical base pair G4•U32 of RNA **2** by the canonical base pair U4-A32 (RNA **3**, Table 1 and Figure 4B) resulted in only minor changes of the binding regions 0–III, but the fraction of complexes with neomycin B binding to the minor groove and overhang decreased to ∼30%, and the fraction of complexes with neomycin B binding to the loop and bulge motif increased to ∼70% (Figure 4A). Further, the introduction of a CUG/CUG motif^97^ with a noncanonical U4•U32 base pair in the lower stem (RNA **4**, Table 1 and Figure 4B) extended binding region 0 to U4 and increased the fraction of complexes with neomycin B binding to the minor groove and overhang to ∼60% (Figure 4A). For RNA **2**, we also studied complexes with two neomycin B molecules and found that the binding regions were the same as in the complexes with one neomycin B molecule (Figure S7). These findings are consistent with the proposed role of noncanonical base pairs in RNA recognition and binding of aminoglycoside ligands^15,35^ and show that CAD MS can be used to detect changes in the location of ligand binding sites as well as for the determination of relative populations of different binding motifs in a given RNA-ligand complex. Finally, to get an idea about how the different binding motifs of RNAs **2**–**4** affect the overall binding affinity for neomycin B, we performed competitive ESI MS binding experiments using RNA **1** as a reference (method 2 in the Supporting Information). The dissociation constants for the complexes of neomycin B with RNAs **2**–**4** were the same within error limits and did not reflect the subtle differences in the occupation of binding motifs A and B derived from CAD data (Table 2), but the second binding motif of RNAs **2**–**4** somewhat increased the overall affinity for neomycin B when compared to RNA **1**.

**Table 2:** *K*_D_ Values for Complexes of Neomycin B with RNA **1** (Calculated by Using Method 1 in the Supporting Information) and RNAs **2**–**4** (Calculated by Using Method 2 and the *K*_D_ of RNA **1** as a Reference)^98,99^ from ESI Spectra (9:1 H_2_O/CH_3_OH at pH ∼7.5, 1 μM RNA Each, 1 μM Neomycin B, 0.25 mM Piperazine, and 50 mM Ammonium Bicarbonate)^a^

RNA	*K*_D_ [μM] at pH 7.5	% motif A	% motif B
**1**	0.7 ± 0.2	100	
**2**	0.5 ± 0.2	∼50	∼50
**3**	0.5 ± 0.2	∼70	∼30
**4**	0.5 ± 0.2	∼40	∼60

a *K*_D_ values are averages from triplicate experiments with errors representing standard deviations including error propagation for RNAs **2**–**4**; occupation of binding motifs A and B from CAD experiments.

## Conclusions

With regard to binding specificity, our native top-down MS data reveal that all neomycin-sensing riboswitch aptamer constructs studied (RNAs **1**–**4**) offer two sequence-distant strand regions for specific binding of neomycin B, referred to here as binding regions I and II. The CAD data for the 27 nt construct designed (and optimized) for NMR studies (RNA **1**) show that neomycin B simultaneously utilizes both regions I and II, which form a single binding pocket for neomycin B (binding motif A), in excellent agreement with NMR studies.^73^ Importantly, in the functionally more relevant 40 nt aptamer constructs (RNA **2**–**4**), we identified two additional binding regions 0 and III (binding motif B). The proportion of complexes with neomycin B binding to motifs A and B and thus the corresponding binding specificities strongly depend on the presence and nature of noncanonical base pairs. For the RNA with the sequence corresponding to the highest regulatory factor for riboswitch function (RNA **2**), we find that binding motifs A and B are equally occupied. Although the biological meaning and relevance of the second binding motif is unclear at this time, one can speculate that the additional binding motif fine-tunes riboswitch regulatory function. For instance, simultaneous occupation of both motifs may impact the repressor/antirepressor stem fold equilibrium, which is responsible for the accessibility of the AUG start codon (nucleotides 38–40) in translational control of riboswitches.

We further stress that a major strength of our FT-ICR MS approach is the experimental ease to investigate sequence mutations. The approach is complementary to NMR spectroscopy and crystallographic approaches and fills a method gap for the determination of middle-to-low affinity RNA small molecule binding sites with the advantage of very little sample amount requirements, no need for stable isotope labeling, and no need for labor-intensive and tedious RNA construct optimization. Any mutation or sequence variation can be easily integrated in the original experimental protocol as demonstrated here by replacing the noncanonical base pair G4•U32 (RNA **2**) in the lower stem with the canonical base pair U4-A32 (RNA **3**), which resulted in decreased binding to motif B. Further, the introduction of a different noncanonical base pair, U4•U32, as part of a CUG/CUG motif in the lower stem (RNA **4**), not only increases the number of nucleotides in the lower stem that interact with neomycin B but also increases binding to motif B. Our data thus show that neomycin B preferentially binds to regions of the riboswitch aptamer constructs that contain noncanonical base pairs^15,35^ and unpaired nucleobases, which also supports the hypothesis of Wöhnert et al. that “ligand specificity of the neomycin riboswitch is encoded at the level of structural dynamics”.^73^ In future studies, we will extend our native top-down mass spectrometry approach to other RNA-aminoglycoside complexes and also investigate new classes of ligands with the ultimate goal to establish general principles of RNA recognition to guide the development of drugs that target RNA.

## Experimental Section

MS experiments were performed at room temperature (21 °C) on a 7 T Fourier transform ion cyclotron resonance (FT-ICR) instrument (Apex Ultra, Bruker, Austria) equipped with an ESI source, a linear quadrupole for ion isolation, a collision cell for CAD, and an ICR cell for ion detection. RNA **1**, sulfate salts of neomycin B and paromomycin, piperidine, piperazine, ammonium acetate, and ammonium bicarbonate were purchased from Sigma-Aldrich (Vienna, Austria). RNAs **2**–**4** were prepared by solid-phase synthesis and purified by HPLC. H_2_O was purified to 18 MΩ·cm at room temperature using a Milli-Q system (Millipore, Austria), and CH_3_OH (VWR, Austria) was HPLC-grade. RNA was desalted by diluting ∼100 μL of RNA solution in H_2_O with ∼400 μL of aqueous ammonium acetate (100 mM) solution followed by concentration to ∼100 μL using centrifugal concentrators (Vivaspin 500, MWCO 3000, Sartorius AG, Germany); the concentration–dilution process was repeated 10 times and followed by 6 cycles of concentration and dilution with H_2_O. The RNA concentration was determined by UV absorption at 260 nm using an Implen nanophotometer (Implen, Germany). ESI spectra were obtained by operation of the linear quadrupole in the transmission mode. For CAD, ions of interest were isolated in the quadrupole and dissociated in the collision cell using the laboratory frame collision energy^92^ indicated in the figure legends. For ESI and CAD, 25–100 and 500 scans were added for each spectrum, respectively. Data reduction utilized the SNAP2 algorithm (Bruker, Austria) or FAST MS, software programmed in our group by Michael Palasser,^34^ as well as manual inspection of the spectra. NMR (at 25 °C) and ITC (at 21 °C) experiments were carried out using a 600 MHz Avance II+ NMR spectrometer equipped with a Prodigy TCI probe (Bruker) and a PEAQ-ITC instrument (MicroCal), respectively.

## Supporting Information Available

(Figure S1) Imino proton NMR spectra; (Figure S2) ITC profiles; (Figure S3) native ESI of RNA **1** with paromomycin; (Figure S4) effect of pH; (Figure S5) effect of collision energy; (Figure S6) effect of complex net charge; (Figure S7) CAD of 1:2 complexes of RNA **2**; calculation of *K*_D_ values from ESI data (PDF)
