# Supplementary material for: Native Top-Down Mass Spectrometry Uncovers Two Distinct Binding Motifs of a Functional Neomycin-Sensing Riboswitch Aptamer
Source: J Am Chem Soc. 2023 Jul 7;145(28):15284–94. doi: 10.1021/jacs.3c02774 (PMC10360057; doi:10.1021/jacs.3c02774)
Supplement: Supplementary file 1 — ja3c02774_si_001.pdf [file ja3c02774_si_001.pdf]

# Native top-down mass spectrometry uncovers two distinct binding motifs of a functional neomycin-sensing riboswitch aptamer

Sarah Viola Heel, Karolina Bartosik, Fabian Juen, Christoph Kreutz, Ronald Micura, and Kathrin Breuker*

Institute of Organic Chemistry and Center for Molecular Biosciences Innsbruck (CMBI), University of Innsbruck, Innrain 80/82, 6020 Innsbruck (Austria)

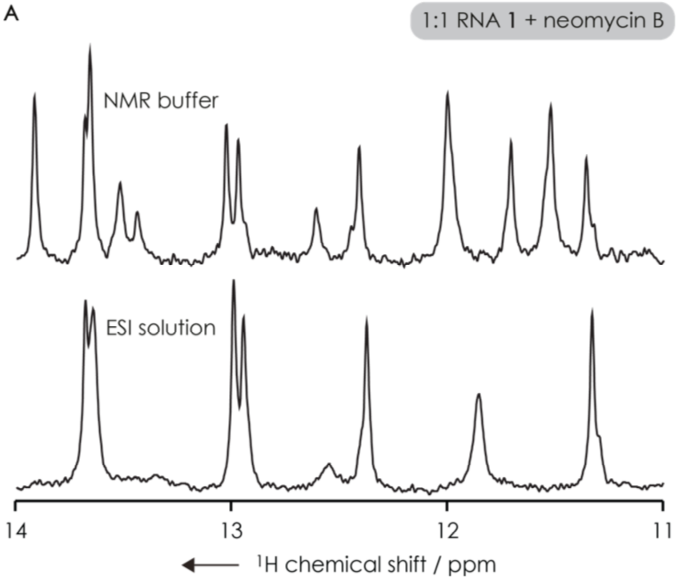


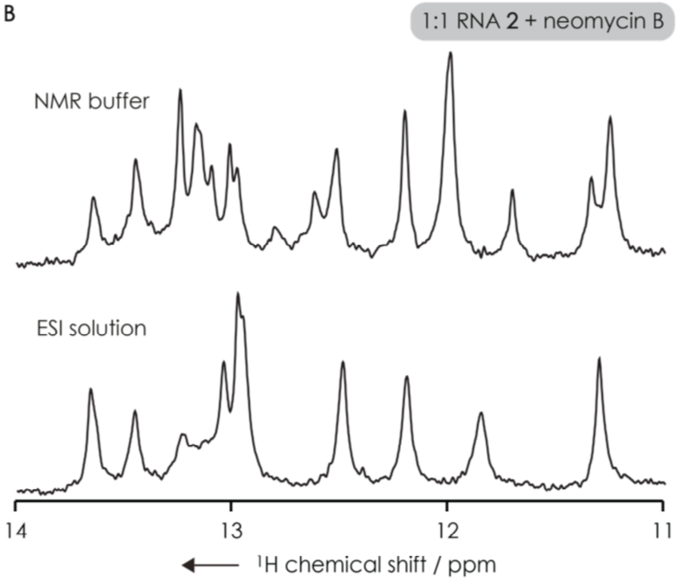


**Figure S1:** $^1$H NMR spectra in the imino proton region for A) RNA **1** and B) RNA **2** with neomycin B (22 μM each) in NMR buffer (15 mM sodium phosphate, 25 mM sodium chloride, 0.1 mM EDTA, 10% vol/vol $D_2O$ with the pH adjusted to 6.5 by addition of sodium hydroxide) and a solution (50 mM ammonium bicarbonate, 10% vol/vol $CD_3OD$, 10% vol/vol $D_2O$, pH 7.5) closely resembling those used for ESI experiments. The imino proton signals provide clear evidence for nucleobase pairing and show that RNAs **1** and **2** are folded in both the NMR buffer and the solution closely resembling those used for ESI experiments. Small differences in chemical shift and peak width between the spectra from the different solutions can be attributed to solvent exchange processes.

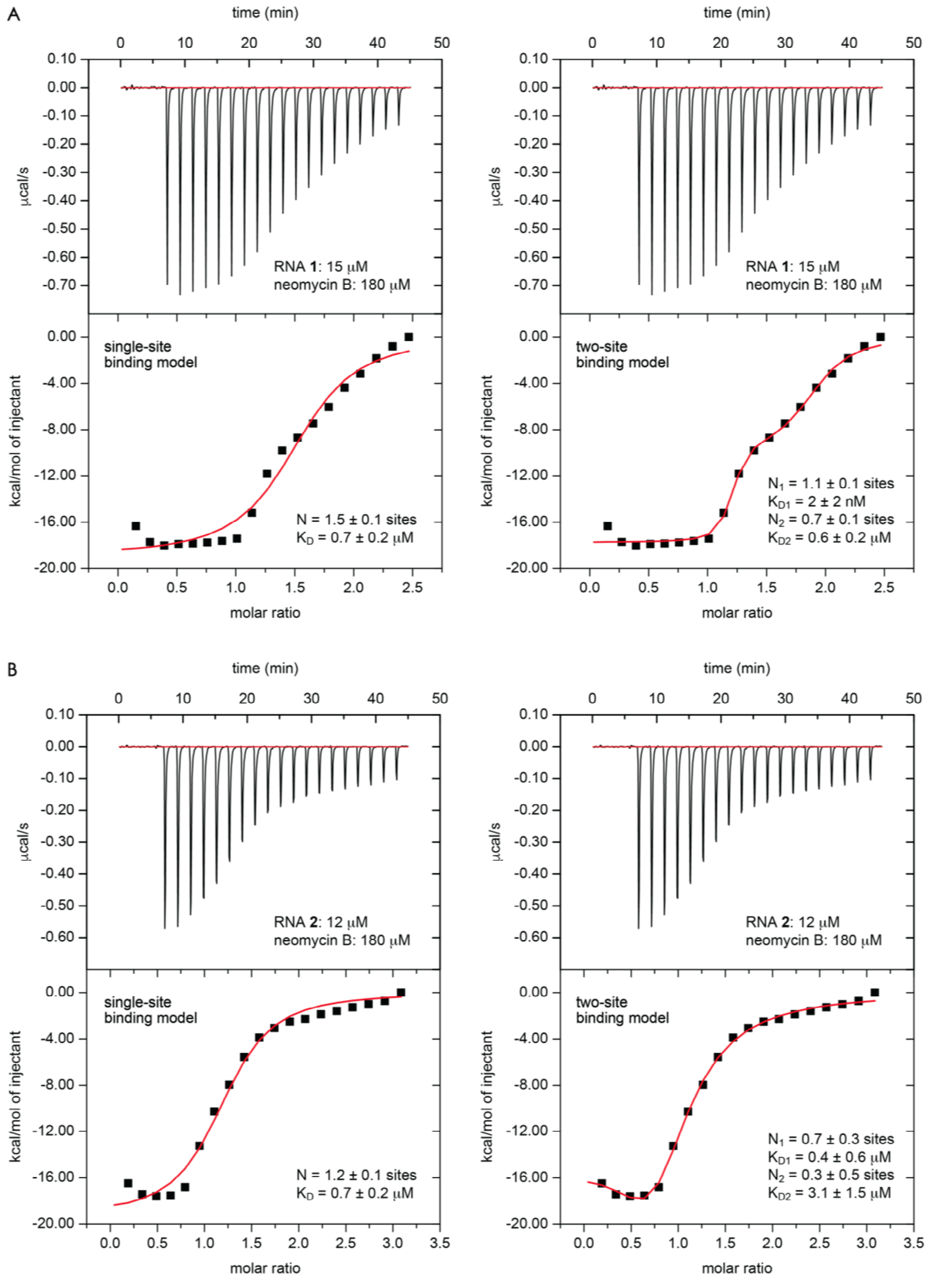


**Figure S2:** ITC profiles for the titration of neomycin B (180 µM) into A) RNA **1** (15 µM) and B) RNA **2** (12 µM) in buffer solution at pH 7.5 (50 mM MOPS, 100 mM potassium chloride, 2 mM magnesium chloride, pH 7.5). Fitting the data using a model for one (left) and two (right) sets of binding sites provides strong evidence for two different binding sites for both RNA **1** and RNA **2**, with the $K_D$ values for RNA **1** differing by ~2.4 orders of magnitude and those for RNA **2** by less than one order of magnitude.

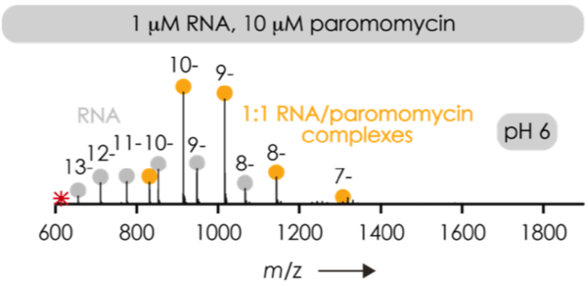


**Figure S3:** Native ESI of RNA **1** (1 μM) and paromomycin (10 μM) in 9:1 $H_2O/CH_3OH$ at pH ~6 (with 0.25 mM piperazine as ESI additive) incubated for 3 hours shows signals of 1:1 complexes (orange circles), free RNA (gray circles) and paromomycin (star).

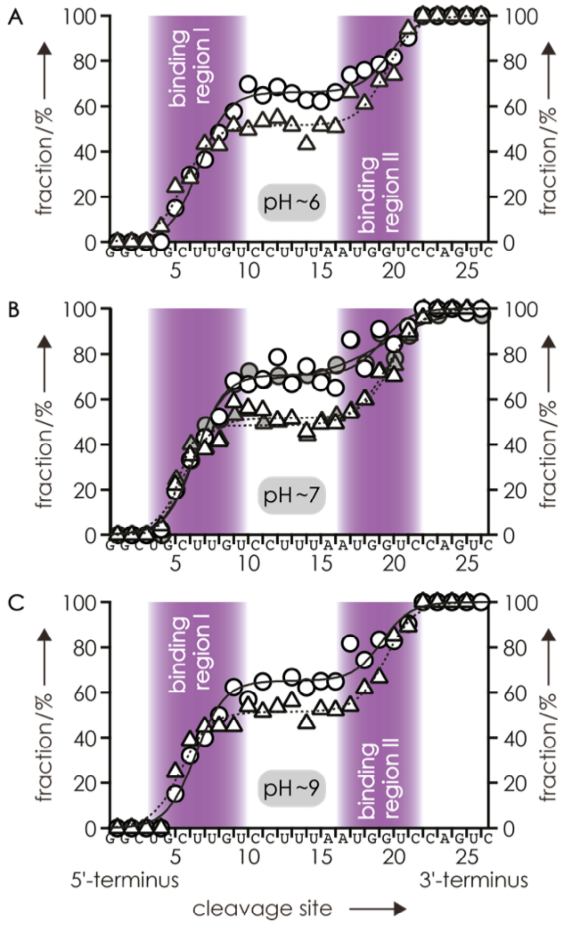


**Figure S4.** Fraction of ***c*** fragments (circles) with and ***y*** fragments (triangles) without neomycin B attached from CAD (103.5 eV laboratory frame energy) of 1:1 complexes of RNA **1** and neomycin B with a net charge of 9-, (RNA **1** + neomycin B - 9H)$^{9-}$, from ESI of solutions at A) pH ~6, B) pH ~7, and C) pH ~9 versus RNA cleavage site; solid (***c*** fragments) and dashed (***y*** fragments) lines are double sigmoidal fits. The 1:1 complexes were electrosprayed from solutions in 9:1 $H_2O/CH_3OH$ and A) 0.25 mM piperazine, B) 2 mM piperazine (white symbols) and 0.25 mM piperazine and 50 mM ammonium bicarbonate (pH ~7.5, gray symbols), and C) 2 mM piperidine as ESI additives; RNA **1** and neomycin B concentrations were 1 μM each.

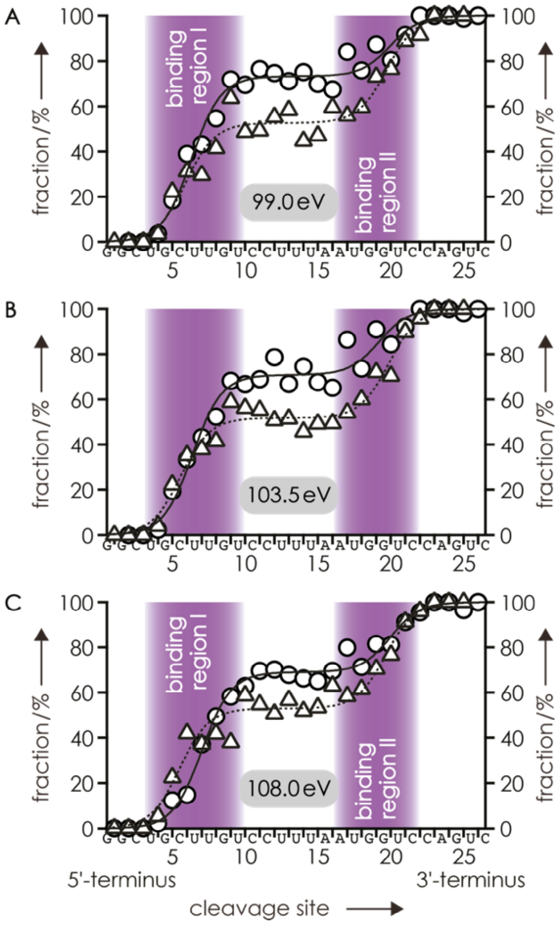


**Figure S5.** Fraction of ***c*** fragments (circles) with and ***y*** fragments (triangles) without neomycin B attached from CAD of 1:1 complexes of RNA **1** and neomycin B with a net charge of 9-, (RNA **1** + neomycin B - 9H)$^{9-}$, from ESI of solutions at pH ~7 (9:1 $H_2O/CH_3OH$, 2 mM piperazine, RNA **1** and neomycin B concentrations were 1 μM each) and laboratory frame energies of A) 99.0 eV, B) 103.5 eV, and C) 108.0 eV versus RNA cleavage site; solid (***c*** fragments) and dashed (***y*** fragments) lines are double sigmoidal fits.

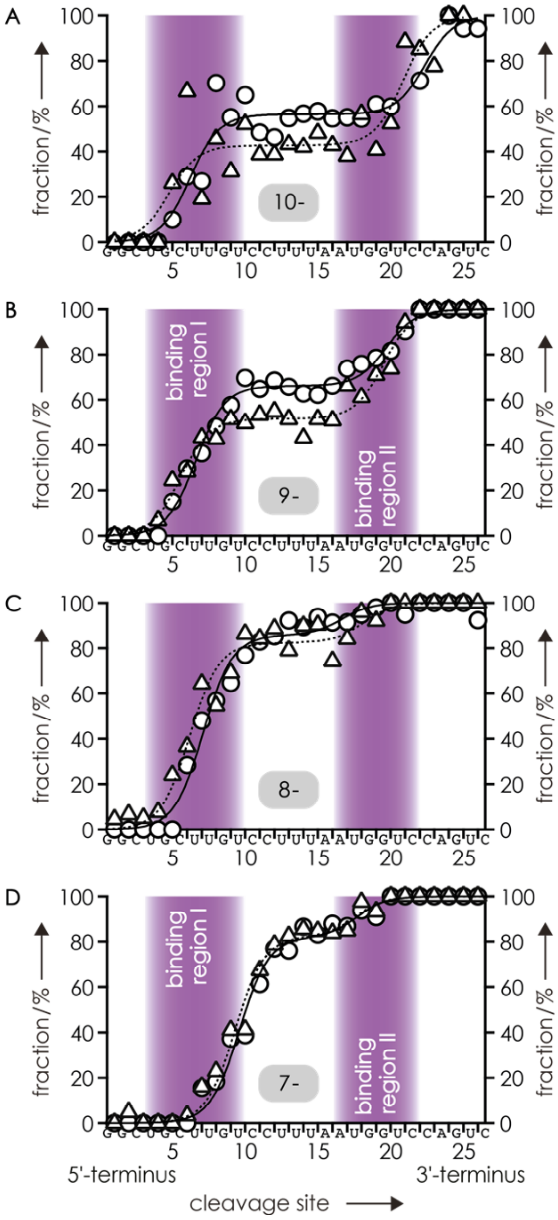


**Figure S6.** Fraction of ***c*** fragments (circles) with and ***y*** fragments (triangles) without neomycin B attached from CAD of 1:1 complexes of RNA **1** and neomycin B for A) (RNA **1** + neomycin B - 10H)$^{10-}$, B) (RNA **1** + neomycin B - 9H)$^{9-}$, C) (RNA **1** + neomycin B - 8H)$^{8-}$, and D) (RNA **1** + neomycin B - 7H)$^{7-}$ ions from ESI of solutions at pH ~6 (9:1 $H_2O/CH_3OH$, 0.25 mM piperazine, RNA **1** and neomycin B concentrations were 1 μM each), versus RNA cleavage site; solid (***c*** fragments) and dashed (***y*** fragments) lines are double sigmoidal fits. The laboratory frame energy for CAD was A) 96 eV, B) 103.5 eV, C) 104 eV, and D) 94.5 eV.

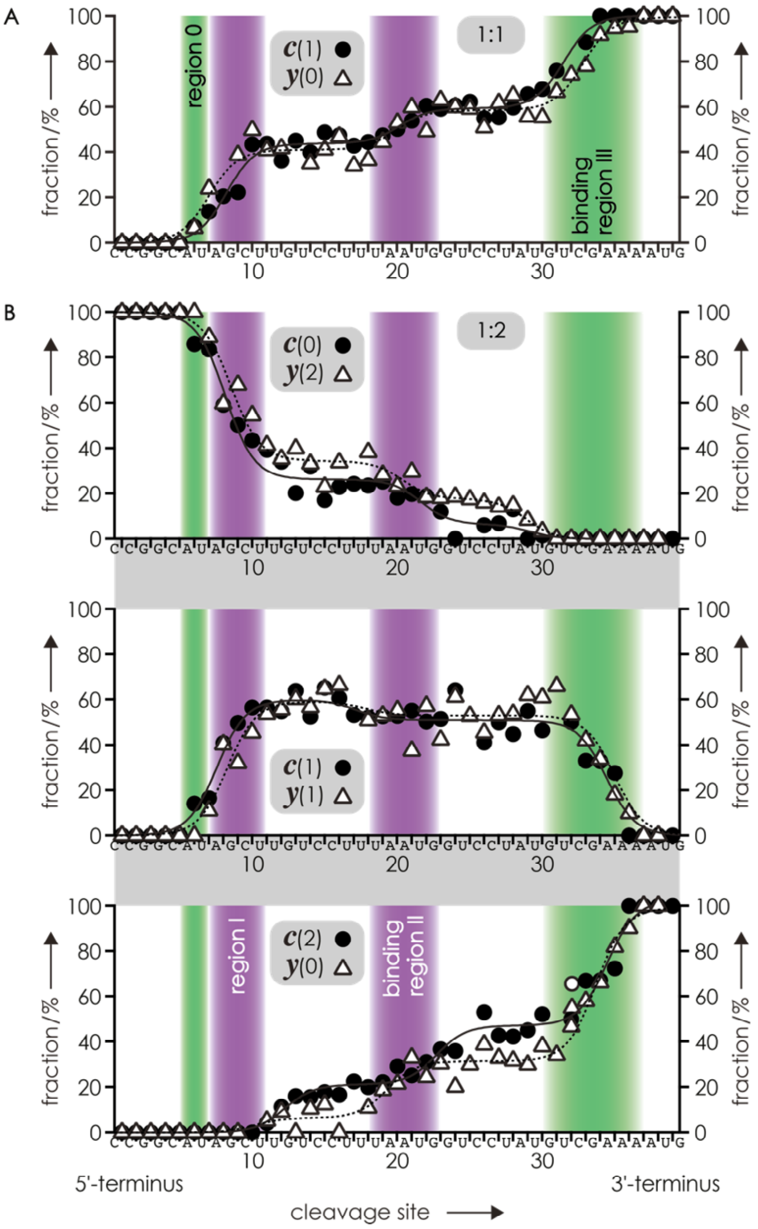


**Figure S7.** Fraction of *c* fragments (circles) and *y* fragments (triangles) with 0 (*c*(0), *y*(0)), 1 (*c*(1), *y*(1)), and 2 (*c*(2), *y*(2)) neomycin B molecules attached as indicated from CAD of A) 1:1 complexes of RNA **2** and neomycin B, (RNA **2** + 1·neomycin B - 13H)$^{13-}$ (this is the same data as in Figure 4A shown for comparison), and B) 1:2 complexes of RNA **2** and neomycin B, (RNA + 2·neomycin B - 13H)$^{13-}$, versus RNA cleavage site; solid (*c* fragments) and dashed (*y* fragments) lines are triple sigmoidal fits. The 1:2 complexes were electrosprayed from solutions at pH ~7.5 (9:1 $H_2O/CH_3OH$, 0.25 mM piperazine and 50 mM ammonium bicarbonate, 1 μM RNA **2**, 2 μM neomycin B).

**$K_D$ calculation from ESI spectra** assumes that the ionization efficiency for RNA and RNA-ligand complexes is the same, and that the relative abundances of RNA and RNA-ligand complex ions calculated from signals in the mass spectra reflect the relative abundances in solution.

method 1 (single equilibrium)

for the reaction $RNA + L \leftrightarrow RNA \bullet L$ the dissociation constant $K_D$ is

$$K_D = \frac{[RNA] \cdot [L]}{[RNA \bullet L]}$$

and the total RNA and ligand concentrations, $[RNA]_0$ and $[L]_0$, respectively, can be written as

$$[RNA]_0 = [RNA] + [RNA \bullet L]$$
$$[L]_0 = [L] + [RNA \bullet L]$$

for an equimolar solution with

$[L]_0 = [RNA]_0$ for which $[L] = [RNA]$

the $K_D$ can be calculated from

$$K_D = \frac{[RNA]^2}{[RNA \bullet L]}$$

method 2 (equilibrium of competitive reactions)

for the competitive reaction of a ligand binding to an RNA under study ($RNA_x$) and a reference RNA ($RNA_{ref}$) with known $K_{D,ref}$

$$RNA_x \bullet L + RNA_{ref} \leftrightarrow RNA_x + L + RNA_{ref} \leftrightarrow RNA_x + RNA_{ref} \bullet L$$

and an equimolar solution with

$$[L]_0 = [RNA_x]_0 = [RNA_{ref}]_0$$

$K_{D,x}$ can be calculated[1,2] from

$$\frac{K_{D,x}}{K_{D,ref}} = \frac{\frac{[RNA_x]}{[RNA_x \bullet L]}}{\frac{[RNA_{ref}]}{[RNA_{ref} \bullet L]}}$$

(1) Wang, W.; Kitova, E. N.; Klassen, J. S. Influence of solution and gas phase processes on protein-carbohydrate binding affinities determined by nanoelectrospray Fourier transform ion cyclotron resonance mass spectrometry. *Anal. Chem.* **2003**, *75*, 4945-4955.

(2) Jørgensen, T. J. D.; Roepstorff, P.; Heck, A. J. R. Direct determination of solution binding constants for noncovalent complexes between bacterial cell wall peptide analogues and vancomycin group antibiotics by electrospray ionization mass spectrometry. *Anal. Chem.* **1998**, *70*, 4427-4432.
